# Role of turnover, downsizing, overtime and night shifts on workplace violence against healthcare workers: a seven-year ecological study

**DOI:** 10.1186/s12889-024-20898-8

**Published:** 2024-12-04

**Authors:** Emanuele M. Giusti, Giovanni Veronesi, Hannah Forest, Monica Ghelli, Benedetta Persechino, Rossana Borchini, Nicola Magnavita, Marco Mario Ferrario

**Affiliations:** 1 EPIMED Research Center, Department of Medicine and Surgery, University of Insubria, Varese, Italy; 2 Department of Occupational and Environmental Medicine, Epidemiology and Hygiene, Italian Workers Compensations Authority (INAIL), Roma, Italy; 3 Occupational and Preventive Medicine, Azienda Socio-Sanitaria Territoriale Lariana, Como, Italy; 4 Università Cattolica del Sacro Cuore, Rome, Italy

**Keywords:** Workplace violence, Personnel turnover, Personnel Downsizing, Night Shift Work, Workload, Health personnel

## Abstract

**Background:**

About one-third of workers identify organisational factors as contributors to workplace violence (WPV), but the associations between these factors and WPV have primarily been explored retrospectively and with measures of perceived organisational constraints, hence providing limited information for prevention. Therefore, we assessed whether objectively measured ward-level indicators of turnover, downsizing, overtime, and night shifts are associated with the occurrence of WPV and whether these associations vary by ward type.

**Methods:**

We conducted an ecological study at a university hospital in northern Italy from 2016 to 2022, using wards as statistical unit (average: 230 wards per year). Active surveillance of WPV was based on an in-hospital incident reporting procedure, updated in November 2021 based on Health Regional Administration guidelines; 2021 was therefore excluded as a transition year. Individual-level administrative data were used to compute ward-level yearly indicators of turnover, downsizing, overtime and night shifts per active worker. Using generalised linear models, we estimated rate ratios (RRs) for yearly WPV counts per 1 SD increase in the indicators, controlling for study period, ward type (emergency department [ED], psychiatric ward, other) and ward sociodemographic composition.

**Results:**

A total of 337 WPV episodes occurred in the 1381 ward-year observations. The WPV rates per 100 active workers per year increased from 1.40 (95%CI: 1.23–1.60) during 2016–2020 to 3.48 (2.90–4.17) in 2022. Higher turnover (RR, 95%CI: 1.47, 1.23–1.75) and downsizing (1.12, 1.00-1.24) were associated with a greater occurrence of WPV across all wards; these associations were consistent across the study periods. In wards with night shift scheduling, turnover (1.64, 1.40–1.92), downsizing (1.21, 1.04–1.40) and the mean number of night shifts (2.50, 1.37–4.56) were associated with WPV. The association between night shifts and WPV was greater in psychiatric wards (RR = 8.73; interaction p-value = 0.02), whereas the role of downsizing was greater in EDs (RR = 1.42, interaction p-value = 0.09) and the role of turnover was greater in the other wards (RR = 1.34, interaction p-value = 0.16).

**Conclusion:**

Work organisational factors are associated with the occurrence of WPV episodes against healthcare workers. Ward type-tailored priorities should be given to minimising turnover and downsizing and promoting a fairer allocation of night shifts to decrease WPV occurrence.

**Supplementary Information:**

The online version contains supplementary material available at 10.1186/s12889-024-20898-8.

## Introduction

Type II workplace violence (WPV) against healthcare workers (HCWs) refers to instances of physical or verbal aggression, threats, intimidation, or sexual harassment perpetrated by patients, their relatives or visitors [1]. This form of violence is a public health concern due to its detrimental impact on the safety, physical and mental well-being of HCWs and the quality of care they provide, leading to the decision to leave the profession [2, 3]. Its rate varies widely depending on the methodology used to record it, which may involve passive retrospective surveys based on the recall of WPV episodes, often conducted on self-selected or at-risk samples, or active surveillance systems that have the advantage of covering the entire workforce. In the latter, the 12-month rate is approximately 2–3 per 100 workers, with higher levels observed in emergency departments (EDs) and psychiatric wards [4, 5]. The most studied risk factors are those related to the offender, such as a history of mental health conditions and altered mental state, and to the victim, such as being a nurse, being male and having less work experience [6–9]. In addition, work organisational factors may significantly contribute to the occurrence of WPV episodes by creating conditions that facilitate them [7]. Indeed, a previous study based on data from an active surveillance system revealed that healthcare workers identified organisational factors as contributing elements in approximately 31% of WPV incident reports [4]. However, despite their potential impact, these factors are less frequently studied and often with inconsistent methodologies.

Work organisational factors that might play a role are related to staffing issues, defined as “insufficient personnel” [7, 10], “personnel cutbacks” [11], or turnover, as well as to factors such as high workload, overtime [12], shift work [13, 14], and frequent night shifts [15]. While these issues may arise from different causes, they share a common link as they are shaped by management decisions regarding worker allocation and scheduling, as well as the psychosocial work environment, which can lead to absenteeism and turnover, increasing the workload for those who remain [16]. These aspects might facilitate WPV episodes since they deplete resources to meet patient demands, increase wait times, and reduce security and organisational support [7]. However, their role in WPV has generally been explored through self-report methods that are susceptible to recall bias and are based on workers’ perceptions (e.g. what constitutes “personnel cutbacks”), with definitions varying across studies. In addition, studies on this topic are often retrospective, which introduces the risk that HCWs who have experienced violence may describe their work environment more negatively. These shortcomings undermine the robustness of the evidence, resulting in limited information for developing effective prevention strategies.

To provide more sound evidence for prevention it is instead important to collect objective measures (e.g., turnover and downsizing on the basis of actual personnel movement rather than on HCW perceptions). In addition, the impact of work organisational factors unfolds over time and is related to the dynamic status of the workforce, with its composition continuously changing due to hirings, terminations and absences. Conducting cross-sectional assessments at a single point in time, on the basis of the registered workers at that moment, fails to account for the fact that each worker’s contribution to the overall active time in a ward varies owing to factors such as long-term absences or movement between wards. This approach can lead to an overestimation of the available workforce and a corresponding underestimation of the impact of organisational factors [17]. To address these issues, administrative data can be leveraged to provide an objective and accurate reconstruction of workforce dynamics over time [18, 19].

The objective of this ecological study was to evaluate at the ward level the associations between turnover, downsizing, overtime, night shift scheduling, and the number of night shifts and the occurrence of WPV episodes against HCWs in hospital departments, adopting an objective and accurate method to assess the exposures from administrative data. In addition, this study aimed to assess these relationships in wards with night shifts, and to assess whether these associations vary by ward type.

## Methods

This ecological seven-year study based on wards as statistical units stems from the “Determinants of Violence against Health Care Workers” (Determinanti Violenze Operatori Sanitari [DeVOS]) project, a multiphase initiative aimed at designing and implementing a guideline-based incident reporting protocol for WPV in two publicly funded referral hospitals in the Lombardy Region [4]. Briefly, the project consisted of the retrospective collection of available WPV data before its start (2016–2020), and the implementation of a new WPV surveillance system, which became effective in November 2021. Among its specific objectives, DeVOS aims to assess the associations between work organisational factors and WPV occurrence. To answer this question, we designed an ecological study using ward-level aggregated yearly information on WPV counts and on indicators of work organisational factors between 2016 and 2022. We excluded 2021 as a transition year between the existing and the new registration standards. In addition, we included only one of the two referral hospitals, namely the Azienda Socio-Sanitaria Territoriale (ASST) Sette Laghi, owing to the lack of consistent ward indications on the retrospective WPV data for the other hospital.

The ASST Sette Laghi is a referral hospital that consists of hub-and-spoke hospitals, rehabilitation hospitals, hospitals dedicated to mothers and children, and outpatient clinics. We included all the medical and surgical units and EDs from spokes and other facilities that were part of the ASST organisation for the entire study period. The DeVOS project received approval from the Ethical Committee of Insubria (ID 82/2021).

### Workplace violence registration

The outcome of this study was the count of WPV per ward. WPV was defined as any occurrence of verbal abuse, threats, physical assaults (directed at individuals or property), or sexual harassment occurring at the workplace and perpetrated by patients and visitors [1].

During the study period, an active WPV monitoring system was in use throughout the entire ASST. This was achieved through the affected worker submitting an incident reporting notification to the hospital risk management office within 48 h of the episode. The form for incident reporting changed in November 2021, with the implementation of the new monitoring standard. Time changes in WPV underreporting and characteristics of the reported violence were documented in a previous study, which found that the ratio of observed to expected WPV episodes based on the literature increased from below 1 prior to the implementation of the DeVOS project to 1.14, i.e. 14% higher than expected from the literature, and that the active monitoring was able to capture both severe WPV episodes (e.g., causing a work injury) and less severe ones (e.g., verbal abuse without co-occurrence of other violence types) [4].

### Definition of the exposure variables

Work organisational factors were investigated via administrative datasets through an epidemiologically-oriented methodology [17], locally developed to assess basic working stress conditions in large working populations, taking into consideration the method proposed by the Italian National Institute for Insurance against Accidents at Work (INAIL) [20]. The starting datasets, provided by the ASST management, included individual-level data of full- and part-time hospital employees, with temporary and permanent contracts, but excluded external consultants. The available information included sociodemographic data, complete employment history with corresponding wards, absences, shift schedules, number of hours worked, hours of absence and hours due by contract for the investigated years. For every worker, we computed the active time at work as the sum of worked days, thus excluding periods of absence due to vacation, sickness, unpaid and maternity leaves, and daily leaves, separately for each ward in which the HCW had worked during the year. This allowed us to accurately reconstruct the active work history of each worker for the years considered in a dynamic fashion (Figure S1, panel A, Supplementary material 1). For each ward and year (e.g., Ward 1 in 2016, Ward 1 in 2017, Ward 2 in 2016, and so on), we then summed the active time at work of all HCW employed in that ward during the given year to calculate the mean number of active workers per ward-year (Figure S1, panel B, Supplementary material 1). For each ward-year observation, the mean number of active workers is interpretable as the daily average over the given year of active workers in the given ward, and it corresponds to approximately 75-80% of the number of workers in the payroll archives on any single day of the year (Supplementary Table S1, Supplementary material 1). The mean number of active workers per ward-year was used as the denominator for calculating the work organisational factors.

For each ward and year, turnover was defined as the sum of new hires and work cessations, including internal transfers between hospital wards, divided by active workers. Downsizing was defined as the difference between work cessations and new hires, also divided by active workers; greater downsizing indicates a net loss of workers in the ward. The overtime indicator was calculated as (hours worked + hours of absence - hours due by contract), divided by active workers, and it was expressed as weekly hours per active worker per year. Finally, we calculated the average number of night shifts per active worker engaged in night shifts per year. Wards with at least two night shifts per month per active worker engaged in night shifts were flagged as having a regular night shift schedule. Finally, from the payroll archives, for each ward we computed the prevalence of female workers, the median age and the median work seniority on June 30th of each year.

### Statistical analysis

We excluded very small (below 3.5 active workers per year; approximately 70 per year) ward-year observations, due to the instability of turnover and downsizing indicators in such small units. The final dataset included 223 wards in 2016, 228 wards in 2017, 235 wards in 2018, 233 wards in 2019, and 231 wards in 2020 and 2022. Due to the skewness of their distributions, we summarised the socio-demographic characteristics and work organisational factors by ward and year using medians and interquartile ranges, in the overall sample first, and then by number of yearly WPV episodes, categorised as 0, 1, and 2 or more.

We then analysed the association between work organisational factors and WPV rates via generalised linear models, with a log link function and assuming negative binomial distributions for the yearly WPV counts to account for the high frequency of wards with zero episodes. Given that the same wards were examined over multiple years, we used a model for repeated measures, specifying an unstructured correlation matrix. The number of active workers per year was used as an offset, while ward type (ED, psychiatric, other), the prevalence of women, median age and median seniority in the ward were covariates. We standardised continuous covariates and work organisational factors using year-specific means and standard deviations as we expected that the SARS-CoV-2 pandemic could have affected their yearly distribution. Hence, the results of the generalised linear models are reported as rate ratios (RRs) with 95% confidence intervals, representing the change in WPV rates for 1 standard deviation (SD) increase in the exposure variables from the year-specific mean.

We initially fitted separate models using data from 2016 to 2020 and from 2022 to assess potential changes in the associations due to modifications in the WPV incident reporting procedures [4]. We then fitted the models using all available years, further adjusting for a dichotomic study period indicator. The independent variables for these models were turnover, downsizing, weekly overtime and having a regular night shift schedule. We replicated the analyses in the subgroup of wards with a regular night shift schedule, adding to the model the mean number of night shifts per active worker engaged in night shifts. Moreover, we added to this model the interaction between ward type and the work organisational factors that were significant in this analysis.

Finally, as a sensitivity analysis to confirm that turnover and downsizing are associated with WPV occurring later in time, we fitted a model using data from 2017 to 2019, with 1-year lagged turnover and downsizing as predictors, controlling for period, percentage of women, median age, and median seniority. We then compared the estimates obtained from this lagged model with those obtained from a cross-sectional model fitted using the same wards.

The α level in all analyses was set at 0.05. The analyses were performed using SAS, version 9.4 (SAS Institute Inc., Cary, NC, USA; proc GENMOD for the generalised linear models).

## Results

The characteristics of the included wards per year are reported in Table 1. Throughout the study period, wards had a median of approximately 10 active workers per year, with approximately 80% being women and a median age of 49 years. Turnover and overtime peaked in 2020 and 2022, coinciding with the pandemic years. The proportion of wards with regular night shifts decreased from 45.3% in 2016 to 37.2% in 2022, with characteristics similar to those of the entire sample of wards; despite this, the mean number of night shifts remained stable over time (Table S2, Supplementary material 1).

**Table 1:** Number of active workers, percentage of women, median age and hospital seniority, and work organisational factors in the investigated wards during the study years

	Year (number of wards)					
	2016 (*n* = 223)	2017 (*n* = 228)	2018 (*n* = 235)	2019 (*n* = 233)	2020 (*n* = 231)	2022 (*n* = 231)
Average number of active workers	10.5 [6.5, 18.3]	10.8 [6.3, 18.7]	10.6 [5.9, 18.1]	10.5 [6.0, 18.5]	10.1 [6.0, 17.9]	10 [ 5.6, 17.9]
Percentage of women	80.0 [66.2, 93.7]	80.6 [63.4, 92.4]	80.0 [63.1, 93.7]	80.0 [64.7, 93.3]	81.0 [64.3, 93.7]	80.0 [66.3, 94.0]
Median age	49.3 [46.1, 52.3]	49.8 [45.6, 53.1]	49.8 [45.7, 53.3]	49.8 [45.8, 53.3]	49.3 [44.4, 53.3]	48.9 [42.6, 53.7]
Median hospital seniority	18.1 [12.6, 23.8]	17.8 [11.7, 24.8]	17.5 [11.2, 24.8]	17.9 [10.8, 25.5]	14.5 [9.0, 23.4]	14.0 [ 5.9, 22.0]
Turnover, per 100 active workers	7.5 [0.0, 20.0]	25.6 [8.3, 51.5]	27.8 [13.2, 48.8]	14.7 [0.0, 28.3]	21.0 [9.2, 38.7]	48.8 [24.5, 87.1]
Downsizing, per 100 active workers	0.0 [0.0, 1.3]	0.0 [-9.5, 0.6]	0.0 [-4.1, 10.7]	0.0 [-7.0, 6.3]	0.0 [-11.3, 11.1]	0.0 [-11.3, 11.6]
Weekly overtime, per active worker	0.9 [0.4, 1.4]	0.9 [0.4, 1.4]	0.9 [0.4, 1.4]	1.0 [0.4, 1.4]	2.8 [1.3, 4.2]	1.7 [0.8, 2.9]
Number of wards with night shift scheduling^a^, n (%)	101 (45.3)	102 (44.7)	101 (43.0)	90 (38.6)	95 (41.1)	86 (37.2)

Note. Medians [interquartile ranges] are reported, unless otherwise specified

^a^ defined as at least 2 night shifts per month per active worker engaged in night shifts

During the period 2016–2020, 224 WPV episodes were registered, corresponding to a rate of 1.40 (95%CI: 1.23–1.60) per 100 active workers per year. In 2022, the number of reported episodes was 113, corresponding to a rate of 3.48 (95%CI: 2.90–4.17) per 100 active workers per year. In wards with a regular night shift schedule, the number of episodes during 2016–2022 and in 2022 was 136 and 96, corresponding to rates of 1.42 (95%CI: 1.20–1.68) and 5.42 (95%CI: 4.46–6.58) per 100 active workers per year, respectively.

Figure S2 (Supplementary material 1) shows the distribution of work organisational factors between ward-year observations with no episodes of WPV, one episode of violence and two or more episodes of violence across the entire study period, considering wards with regular night shift scheduling. Ward-year observations with one or two or more episodes of WPV had higher turnover rates (median = 58.5 and 33.5, respectively) and number of night shifts (median = 62.2 and 63.2, respectively), compared to wards with no WPV episodes (median turnover = 29.4, median number of night shifts = 57.6, *p* < .05). The difference in turnover was observed across the entire study sample, along with a difference in the probability of having a regular night shift schedule (Supplementary Table S3, Supplementary material 1).

### Associations between work organisational factors and WPV rates

The results of the generalised linear models evaluating the associations between work organisational factors and rates of WPV episodes are reported in Table 2. ED wards and psychiatric wards presented higher WPV compared to the other wards. In 2016–2020, turnover was associated with increased WPV rates, whereas downsizing, weekly overtime and night shift scheduling showed no significant associations. In 2022, the association between turnover and WPV rates decreased, and the association between downsizing and WPV rates increased, although both effects were nonsignificant. Across the entire study period, higher turnover (RR, 95%CI: 1.47, 1.23–1.75) and downsizing (RR, 95%CI: 1.12, 1.00–1.24) were associated with increased WPV rates.

**Table 2:** Associations between ward type, percentage of women, median age, hospital seniority, and work organisational factors with the rates of workplace violence episodes, by study years

	2016-2020^a^		2022^b^		2016–2020, 2022^c^	
	RR	95% CI	RR	95% CI	RR	95% CI
Ward type – ED vs. Other wards	5.12	1.42–18.39	19.67	2.95–131.18	8.28	2.90–23.65
Ward type – Psychiatric vs. Other wards	20.86	5.18–84.02	5.44	1.32–22.50	15.95	4.95–51.43
Percentage of women	1.19	0.68–2.10	0.51	0.28–0.95	0.90	0.59–1.38
Median age	1.77	0.87–3.58	1.33	0.53–3.36	1.51	0.84–2.70
Median hospital seniority	0.51	0.27–0.97	0.85	0.32–2.24	0.61	0.35–1.08
Turnover	1.46	1.17–1.83	1.05	0.44–2.50	1.47	1.23–1.75
Downsizing	1.09	0.90–1.33	1.30	0.42–4.06	1.12	1.00–1.24
Weekly overtime	0.71	0.42–1.21	0.59	0.23–1.52	0.73	0.53–1.02
Night shift scheduling	1.03	0.44–2.37	1.00	0.31–3.28	0.97	0.49–1.93
Period – 2022 vs. 2016–2020	-	-	-	-	2.76	1.43–5.30

Note. Coefficients represent change in WPV rates for 1 SD increase from the year-specific mean. All coefficients are mutually adjusted. Abbreviations: WPV: Workplace Violence; RR: Rate ratio; CI: Confidence interval; ED: Emergency Department

^a^*n* = 1150 ward-year observations, 224 WPV episodes, WPV rate: 1.04 (95%CI 1.23–1.60) per 100 active workers per year

^b^*n* = 231 ward-year observations, 113 WPV episodes, WPV rate: 3.48 (95%CI: 2.90–4.17) per 100 active workers per year

^c^*n* = 1381 ward-year observations, 337 WPV episodes, WPV rate: 1.75 (95%CI: 1.57–1.94) per 100 active workers per year

Table 3 reports the results of the analysis performed in the subgroup of wards with a regular night shift schedule. Over the entire study period, turnover (RR, 95%CI: 1.64, 1.40–1.92), downsizing (RR, 95%CI: 1.04–1.40), and the number of night shifts (RR, 95%CI: 2.50, 1.37–4.56) were associated with higher WPV rates.

**Table 3:** Associations between work organisational factors and rates of workplace violence episodes in wards with night shift scheduling over the entire study period

	RR	95% CI
Ward type – ED vs. Other wards	4.97	1.83–13.51
Ward type – Psychiatric vs. Other wards	6.95	2.51–19.23
Percentage of women	0.87	0.53–1.44
Median age	2.96	1.94–4.49
Median hospital seniority	0.23	0.11–0.50
Turnover	1.64	1.40–1.92
Downsizing	1.21	1.04–1.40
Weekly overtime	1.24	0.75–2.06
Number of night shifts^a^	2.50	1.37–4.56
Period – 2022 vs. 2016–2020	2.96	1.30–6.73

Note. Coefficients represent change in WPV rates for each one standard deviation increase from the year-specific mean. All coefficients are mutually adjusted. The model was run using data from all available years (2016–2020 and 2022, *n* = 575 ward-year observations, 232 WPV episodes, WPV rate: 2.05 [95%CI: 1.08–2.32] per 100 active workers per year). Abbreviations: RR: Rate ratio. CI: Confidence interval, ED: Emergency Department

^a^ Per active worker engaged in night shifts

In the models including the interaction terms (Table 4), the interaction between the mean number of night shifts and ward type was significant, indicating that the mean number of night shifts had a stronger association with WPV rates in psychiatric wards (RR, 95%CI: 8.73, 1.92–39.70) than in the ED or in the other wards. The association between downsizing and WPV was stronger in EDs (RR, 95%CI: 1.42, 0.76–2.68), whereas the association between turnover and WPV was stronger in the other wards (RR, 95%CI: 1.34, 1.18–1.53). However, these interactions were not statistically significant.

**Table 4:** Interactions between turnover, downsizing, number of night shifts and ward type in wards with night shift scheduling over the entire study period

	RR	95% CI	Interaction *p*-value
Turnover (ED)	0.77	0.42–1.41	0.16
Turnover (Psychiatric wards)	0.33	0.09–1.14	
Turnover (Other wards)	1.34	1.18–1.53	
Downsizing (ED)	1.42	0.76–2.68	0.09
Downsizing (Psychiatric wards)	0.32	0.10–1.02	
Downsizing (Other wards)	1.05	0.94–1.17	
Number of night shifts^a^ (ED)	1.62	0.36–7.26	0.02
Number of night shifts^a^ (Psychiatric wards)	8.73	1.92–39.70	
Number of night shifts^a^ (Other wards)	1.36	0.70–2.63	

Note. Coefficients represent change in WPV rates for each one standard deviation increase from the year-specific mean. All coefficients are mutually adjusted. The models are also adjusted for period, weekly overtime, female percentage, median age, median seniority. The model was run using data from all available years (2016–2020 and 2022, *n* = 575 ward-year observations, 232 WPV episodes, WPV rate: 2.05 [95%CI: 1.08–2.32] per 100 active workers per year). Abbreviations: RR: Rate ratio. CI: Confidence interval, ED: Emergency Department

^a^ Per active worker engaged in night shifts

The sensitivity analysis revealed that 1-year lagged turnover maintained a significant, although attenuated compared to turnover measured the same year, association with WPV (cross-sectional model: RR, 95%CI: 2.14, 1.58–2.90; 1-year lagged model: RR, 95%CI = 1.35, 1.03–1.78). Downsizing exhibited similar associations in both models (cross-sectional model: RR, 95%CI: 1.19, 0.96–1.47; 1-year lagged model: RR, 95%CI: 1.18, 0.76–1.81).

## Discussion

In this study, we found that higher turnover and downsizing were associated with increased rates of WPV episodes across all wards, including EDs, in a large referral hospital. In addition, in wards with regular night shifts, turnover, downsizing and the mean number of night shifts per active worker were associated with WPV, with the impact of night shifts being greater in psychiatric wards.

Previous studies have investigated WPV as a predictor of turnover, but generally framed it as turnover intention (i.e., the self-reported likelihood or willingness to leave their current job) and employed cross-sectional designs that make it difficult to draw conclusions about the direction of the association [21, 22]. In line with other studies addressing its negative outcomes, our study adds to the literature suggesting that objectively-measured turnover (i.e., actual personnel movement) may be an organizational factor contributing to WPV. Indeed, previous studies have shown that turnover negatively affects HCWs both at the individual level, by impairing work satisfaction and mental health [23, 24], and at the team level, by reducing team cohesion [23]. In addition, it has a negative impact on patients’ health, reduces their satisfaction with care and increases their length of stay [25–27]. These factors likely contribute to the increased incidence of WPV, as they exacerbate stress and dissatisfaction not only for staff but also for patients and their relatives. Notably, this association persisted even when turnover from the year preceding the WPV episode was considered as a predictor. Our findings indicate that high turnover is associated with higher WPV rates independent of downsizing, suggesting that frequent personnel changes might be harmful in themselves, while downsizing appears to amplify them. In addition to reducing the number of staff available to manage difficult interactions with patients or relatives, downsizing may increase stress and dissatisfaction among HCWs by increasing their workload, lowering job satisfaction, and amplifying burnout and psychological distress [28, 29]. This aspect might be particularly important in EDs because the reduction in personnel has a greater impact in settings with high patient fluxes, making it more challenging to manage interaction with patients and/or visitors with fewer HCWs available.

Turnover and downsizing are influenced by different underlying factors, but managerial interventions can and should effectively address them [30]. On the one hand, studies on risk factors for turnover intention show that low salary levels, a heavier workload, greater dissatisfaction with the organisation, a lack of training, and a higher bed-to-nurse ratio are associated with a greater intention to leave [31, 32]. On the other hand, studies on measures to reduce turnover and downsizing show that these can be mitigated by improving career prospects, increasing recognition, job satisfaction, team support and team relationships, and mitigating workplace bullying [16, 33, 34]. The results of our study suggest that establishing a more stable workforce (i.e., reducing turnover and downsizing), might represent a path for the prevention of WPV.

After taking into account ward type, we did not observe a difference in WPV rates between wards with regular night shifts and those without. However, in wards with regular night shifts, we found that, in addition to the effects of turnover and downsizing, the frequency of night shifts per worker engaged in night shifts is associated with the occurrence of WPV. This suggests that the issue may not be the presence of night shifts per se, but rather their frequency for individual active workers. Increased frequency of night shifts may lead to greater exposure to work periods with fewer staff and patients who are more likely to engage in aggressive behaviour (due, for instance, to their clinical conditions or a substance-related altered state), thereby increasing the probability of WPV [15, 35]. In addition, despite their detrimental effects on physical health due to the disruption of circadian rhythms, night shifts also increase the perception of more demanding or constraining working conditions, disrupt sleep quality and exacerbate the risk of mental health issues, including depression and burnout [36–39]. These issues might reduce HCWs’ psychological resources to effectively manage difficult interactions with patients. Therefore, distributing night shifts more evenly among workers might help preventing WPV.

The association between the frequency of night shifts and WPV appeared stronger in psychiatric wards. Although estimates of WPV rates across different shift times vary among studies, and comparisons are complicated by differing denominators, such as fewer visitors, patients, and healthcare workers during night shifts, previous research has shown that the number of WPV episodes in psychiatric wards is relatively evenly distributed between day and night shifts [40]. Therefore, it remains uncertain whether the observed association in psychiatric wards is due to increased exposure to patients in altered states at night. Another potential explanation could involve reduced HCW resources during night shifts in psychiatric wards, which can lead to increased fatigue and decreased job control, factors that have been recognised as critical in increasing the likelihood of being assaulted [41]. This suggests that addressing patient factors alone, such as the use of pharmacological interventions to reduce night-time agitation, may not be sufficient. To better manage the risks of WPV, providing HCWs on night shifts in psychiatric wards with comprehensive support, such as adequate staffing, training on handling WPV and supportive interventions, might be equally important.

The main strength of this study lies in its use of objective data through a methodology that accounts for the actual active workforce, thus avoiding recall bias and providing a more accurate assessment of work constraints. A limitation of this study is that, while we examined how organisational factors influence WPV, bidirectional relationships between these factors cannot be ruled out, in particular for turnover and downsizing. Although our sensitivity analysis showed that the observed relationships persisted even when using 1-year lagged predictors, further studies with longitudinal designs are needed to clarify which direction of causality is stronger. Second, since this study aggregates data at the ward level, the observed associations between work organisational factors and WPV should not be interpreted as direct causal relationships at the individual worker level to avoid the risk of ecological fallacy. Third, our outcome measures are based on the reported incidence of violence, which is known to be lower than the actual occurrence [42]. However, it should be noted that the new reporting method implemented in the study hospital has significantly reduced underreporting [4], and the associations between work organisational factors and WPV remained substantially consistent both before and after its implementation. Finally, as the study was conducted with data from hospitals of a single institution, the results should be validated in different contexts to confirm their generalizability.

## Conclusions

In conclusion, in this study we found that objectively-measured work organisational factors, in particular turnover, downsizing and frequent night shifts, are associated with the occurrence of WPV episodes against healthcare workers. To mitigate WPV, ward-type tailored priorities should be given to improving workforce retention, for example by focusing on improving career prospects, workers’ recognition, job satisfaction, team support and team relationships. Additionally, efforts should be made to ensure a more balanced distribution of night shifts across HCWs, particularly in psychiatric wards. Providing comprehensive support to workers in wards with frequent night shifts, through mental health support services (e.g., counselling services, stress management programs, and peer support groups), adequate staffing, and strategies to mitigate fatigue and burnout is important for preventing WPV. Our results align with the WHO/ILO Framework guidelines for addressing workplace violence [43] and with the ILO Violence and Harassment Convention, which highlights that promoting decent work is an essential strategy to prevent WPV occurrence and mitigate the public health threat it poses [44].

## Electronic supplementary material

Below is the link to the electronic supplementary material.

Supplementary Material 1

## Data Availability

The data underlying this article cannot be shared publicly due to privacy reasons but are available from the corresponding author on reasonable request.

## Abbreviations

CI: Confidence interval
ED: Emergency Departments
HCW: Healthcare worker
RR: Rate ratio
SD: Standard deviation
WPV: Workplace Violence

## References

[1] National Institute of Occupational Safety and Health. Violence - Occupational hazards in hospitals. 2020 Jun 30 [cited 2024 Jul 26]; https://www.cdc.gov/niosh/docs/2002-101/default.html

[2] Nyberg A, Kecklund G, Hanson LM, Rajaleid K. Workplace violence and health in human service industries: a systematic review of prospective and longitudinal studies. Occup Environ Med. 2021;78(2):69–81. 32414952 10.1136/oemed-2020-106450 PMC7873420

[3] Kuhlmann E, Brînzac MG, Czabanowska K, Falkenbach M, Ungureanu MI, Valiotis G, et al. Violence against healthcare workers is a political problem and a public health issue: a call to action. Eur J Public Health. 2023;33(1):4–5. 36508506 10.1093/eurpub/ckac180 PMC9897982

[4] Veronesi G, Ferrario MM, Giusti EM, Borchini R, Cimmino L, Ghelli M, et al. Systematic violence monitoring to reduce Underreporting and to Better Inform Workplace Violence Prevention among Health Care Workers: before-and-after prospective study. JMIR Public Health Surveill. 2023;9:e47377. 37955961 10.2196/47377 PMC10682923

[5] Viottini E, Politano G, Fornero G, Pavanelli PL, Borelli P, Bonaudo M, et al. Determinants of aggression against all health care workers in a large-sized university hospital. BMC Health Serv Res. 2020;20(1):215. 32178674 10.1186/s12913-020-05084-x PMC7077118

[6] Berger S, Grzonka P, Frei AI, Hunziker S, Baumann SM, Amacher SA, et al. Violence against healthcare professionals in intensive care units: a systematic review and meta-analysis of frequency, risk factors, interventions, and preventive measures. Crit Care. 2024;28(1):61. 38409034 10.1186/s13054-024-04844-z PMC10898135

[7] O’Brien CJ, van Zundert AAJ, Barach PR. The growing burden of workplace violence against healthcare workers: trends in prevalence, risk factors, consequences, and prevention – a narrative review. eClinicalMedicine [Internet]. 2024 Jun 1 [cited 2024 Aug 30];72. https://www.thelancet.com/journals/eclinm/article/PIIS2589-5370(24)00220-7/fulltext 10.1016/j.eclinm.2024.102641 PMC11152903 38840669

[8] Yusoff HM, Ahmad H, Ismail H, Reffin N, Chan D, Kusnin F, et al. Contemporary evidence of workplace violence against the primary healthcare workforce worldwide: a systematic review. Hum Resour Health. 2023;21(1):82. 37833727 10.1186/s12960-023-00868-8 PMC10576303

[9] Taherzadeh Chenani K, Jahangiri M, Madadizadeh F, Sadat Anoosheh V. Factors associated with occurrence of workplace violence against healthcare workers during the COVID-19 pandemic: a meta-analysis. Int J Occup Saf Ergon. 2024;1–9. 10.1080/10803548.2024.2381981 39154295

[10] Mento C, Silvestri MC, Bruno A, Muscatello MRA, Cedro C, Pandolfo G, et al. Workplace violence against healthcare professionals: a systematic review. Aggress Violent Beh. 2020;51:101381.

[11] Viitasara E, Sverke M, Menckel E. Multiple risk factors for violence to seven occupational groups in the Swedish Caring Sector. Relations Indistrielles/Ind Relat. 2003;58.

[12] Kumari A, Ranjan P, Sarkar S, Chopra S, Kaur T, Baitha U. Identifying predictors of Workplace Violence Against Healthcare professionals: a systematic review. Indian J Occup Environ Med. 2022;26(4):207–24. 37033752 10.4103/ijoem.ijoem_164_21 PMC10077728

[13] Tian Y, Yue Y, Wang J, Luo T, Li Y, Zhou J. Workplace violence against hospital healthcare workers in China: a national WeChat-based survey. BMC Public Health. 2020;20:582. 32349727 10.1186/s12889-020-08708-3 PMC7189471

[14] Cheung T, Yip PSF. Workplace violence towards nurses in Hong Kong: prevalence and correlates. BMC Public Health. 2017;17(1):196. https://bmcpublichealth.biomedcentral.com/articles/10.1186/s12889-017-4112-3. 10.1186/s12889-017-4112-3 PMC5310001 28196499

[15] Sun S, Gerberich SG, Ryan AD. The relationship between Shiftwork and Violence Against nurses: a Case Control Study. Workplace Health Saf. 2017;65(12):603–11. 28535713 10.1177/2165079917703409

[16] Mathisen J, Nguyen TL, Jensen JH, Rugulies R, Rod NH. Reducing employee turnover in hospitals: estimating the effects of hypothetical improvements in the psychosocial work environment. Scand J Work Environ Health. 2021;47(6):456–65. 34052852 10.5271/sjweh.3969 PMC8504546

[17] Veronesi G, Bertù L, Mombelli S, Cimmino L, Caravati G, Conti M, et al. [Methodological aspects in the evaluation of turn-over and up/down sizing as indicators of work-related stress]. G Ital Med Lav Ergon. 2011;33(3 Suppl):323–5. 23393867

[18] Lachowska M, Mas A, Woodbury SA. How reliable are administrative reports of paid work hours? Labour Econ. 2022;75:102131.

[19] Vestergaard JM, Haug JND, Dalbøge A, Bonde JPE, Garde AH, Hansen J, et al. Validity of self-reported night shift work among women with and without breast cancer. Scand J Work Environ Health. 2024;50(3):152–7. 38329266 10.5271/sjweh.4142 PMC11006433

[20] INAIL. [La Metodologia per la valutazione e gestione del rischio stress lavoro-correlato]. INAIL; 2017.

[21] Chen M, Xie H, Liao X, Ni J. Workplace violence and turnover intention among Chinese nurses: the mediating role of compassion fatigue and the moderating role of psychological resilience. BMC Public Health. 2024;24(1):2437. 39244556 10.1186/s12889-024-19964-y PMC11380784

[22] Liu W, Zhao S, Shi L, Zhang Z, Liu X, Li L, et al. Workplace violence, job satisfaction, burnout, perceived organisational support and their effects on turnover intention among Chinese nurses in tertiary hospitals: a cross-sectional study. BMJ Open. 2018;8(6):e019525. 29886440 10.1136/bmjopen-2017-019525 PMC6009508

[23] Bae SH, Kim S, Myung H. Mediating effects of workgroup processes on the relationship between nurse turnover and nurse outcomes in hospitals. Front Public Health. 2023;11:1255983. 10.3389/fpubh.2023.1255983. 10.3389/fpubh.2023.1255983 PMC10701376 38074708

[24] Hayes LJ, O’Brien-Pallas L, Duffield C, Shamian J, Buchan J, Hughes F, et al. Nurse turnover: a literature review - an update. Int J Nurs Stud. 2012;49(7):887–905. 22019402 10.1016/j.ijnurstu.2011.10.001

[25] Park SH, Boyle DK, Bergquist-Beringer S, Staggs VS, Dunton NE. Concurrent and lagged effects of registered nurse turnover and staffing on unit-acquired pressure ulcers. Health Serv Res. 2014;49(4):1205–25. 24476194 10.1111/1475-6773.12158 PMC4239846

[26] Husted H, Hansen HC, Holm G, Bach-Dal C, Rud K, Andersen KL, et al. What determines length of stay after total hip and knee arthroplasty? A nationwide study in Denmark. Arch Orthop Trauma Surg. 2010;130(2):263–8. 19633865 10.1007/s00402-009-0940-7

[27] Bae S. Noneconomic and economic impacts of nurse turnover in hospitals: a systematic review. Int Nurs Rev. 2022;69(3):392–404. 35654041 10.1111/inr.12769 PMC9545246

[28] Langster HJ, Cutrer S. A scoping review of the impact of Downsizing on survivors. JONA: J Nurs Adm. 2021;51(6):329. 10.1097/NNA.0000000000001022 33989238

[29] Burke RJ, Ng ES, Wolpin J. Effects of hospital restructuring and downsizing on nursing staff: the role of union support. J Health Manage. 2016;18(3):473–88.

[30] Iverson RD, Pullman JA. Determinants of Voluntary turnover and layoffs in an environment of repeated downsizing following a Merger: an event history analysis. J Manag. 2000;26(5):977–1003.

[31] Wu F, Lao Y, Feng Y, Zhu J, Zhang Y, Li L. Worldwide prevalence and associated factors of nursing staff turnover: a systematic review and meta-analysis. Nurs Open. 2024;11(1):e2097. 38268271 10.1002/nop2.2097 PMC10802134

[32] Kim Y, Kim HY. Retention Rates and the Associated Risk factors of turnover among newly hired nurses at South Korean hospitals: a retrospective cohort study. Int J Environ Res Public Health. 2021;18(19):10013. 34639317 10.3390/ijerph181910013 PMC8507922

[33] Perry J. What can healthcare organisations do to improve medical engagement? A systematic review. BMJ Lead. 2024;leader-2023-000963. 10.1136/leader-2023-000963 39168607

[34] Chen Y, Zhou X, Bai X, Liu B, Chen F, Chang L, et al. A systematic review and meta-analysis of the effectiveness of social support on turnover intention in clinical nurses. Front Public Health. 2024;12:1393024. 38903567 10.3389/fpubh.2024.1393024 PMC11187297

[35] Johnson J, Hansen S, Hopper L, Brook L, Watson J, Mills B. Aggression and violence in the emergency department: a qualitative study exploring the perspectives of frontline healthcare professionals. Collegian. 2024;31(4):195–201.

[36] Wu QJ, Sun H, Wen ZY, Zhang M, Wang HY, He XH, et al. Shift work and health outcomes: an umbrella review of systematic reviews and meta-analyses of epidemiological studies. J Clin Sleep Med. 2022;18(2):653–62. 34473048 10.5664/jcsm.9642 PMC8804985

[37] Giusti EM, Veronesi G, Gianfagna F, Magnavita N, Campana F, Borchini R et al. The independent and interactive effects of changes in overtime and night shifts during the COVID-19 pandemic on burnout among nurses: a longitudinal study. Scand J Work Environ Health. 2024;4176. 10.5271/sjweh.4176 PMC11395668 38970449

[38] Torquati L, Mielke GI, Brown WJ, Burton NW, Kolbe-Alexander TL. Shift work and poor Mental Health: a Meta-analysis of Longitudinal studies. Am J Public Health. 2019;109(11):e13. 31536404 10.2105/AJPH.2019.305278 PMC6775929

[39] Harris R, Kavaliotis E, Drummond SPA, Wolkow AP. Sleep, mental health and physical health in new shift workers transitioning to shift work: systematic review and meta-analysis. Sleep Med Rev. 2024;75:101927. 38626702 10.1016/j.smrv.2024.101927

[40] Weltens I, Bak M, Verhagen S, Vandenberk E, Domen P, van Amelsvoort T et al. Aggression on the psychiatric ward: prevalence and risk factors. A systematic review of the literature. PLoS ONE. 2021;16(10). 10.1371/journal.pone.0258346 PMC8500453 34624057

[41] Niu SF, Kuo SF, Tsai HT, Kao CC, Traynor V, Chou KR. Prevalence of workplace violent episodes experienced by nurses in acute psychiatric settings. PLoS ONE. 2019;14(1):e0211183. 30677077 10.1371/journal.pone.0211183 PMC6345477

[42] Phillips JP. Workplace Violence against Health Care Workers in the United States. N Engl J Med. 2016;374(17):1661–9. 27119238 10.1056/NEJMra1501998

[43] ILO/ICN/WHO/PSI Joint Programme on Workplace Violence in the Health Sector. Framework guidelines for addressing workplace violence in the health sector / Joint Programme on Workplace Violence in the Health Sector [Internet]. Geneva. 2002. https://iris.who.int/handle/10665/42617

[44] International Labour Organization. C190 - Violence and Harassment Convention, 2019 [Internet]. 2019 [cited 2024 Sep 24]. https://normlex.ilo.org/dyn/normlex/en/f?p=NORMLEXPUB:12100:0::NO::P12100_ILO_CODE:C190

