# Supplementary material for: Role of turnover, downsizing, overtime and night shifts on workplace violence against healthcare workers: a seven-year ecological study
Source: BMC Public Health. 2024 Dec 4;24:3371. doi: 10.1186/s12889-024-20898-8 (PMC11616121; doi:10.1186/s12889-024-20898-8)
Supplement: Supplementary file 1 — Supplementary Material 1 [file 12889_2024_20898_MOESM1_ESM.docx]

**Supplementary material 1 -**

**Role of turnover, downsizing, overtime and night shifts on workplace violence against healthcare workers: a seven-year ecological study**


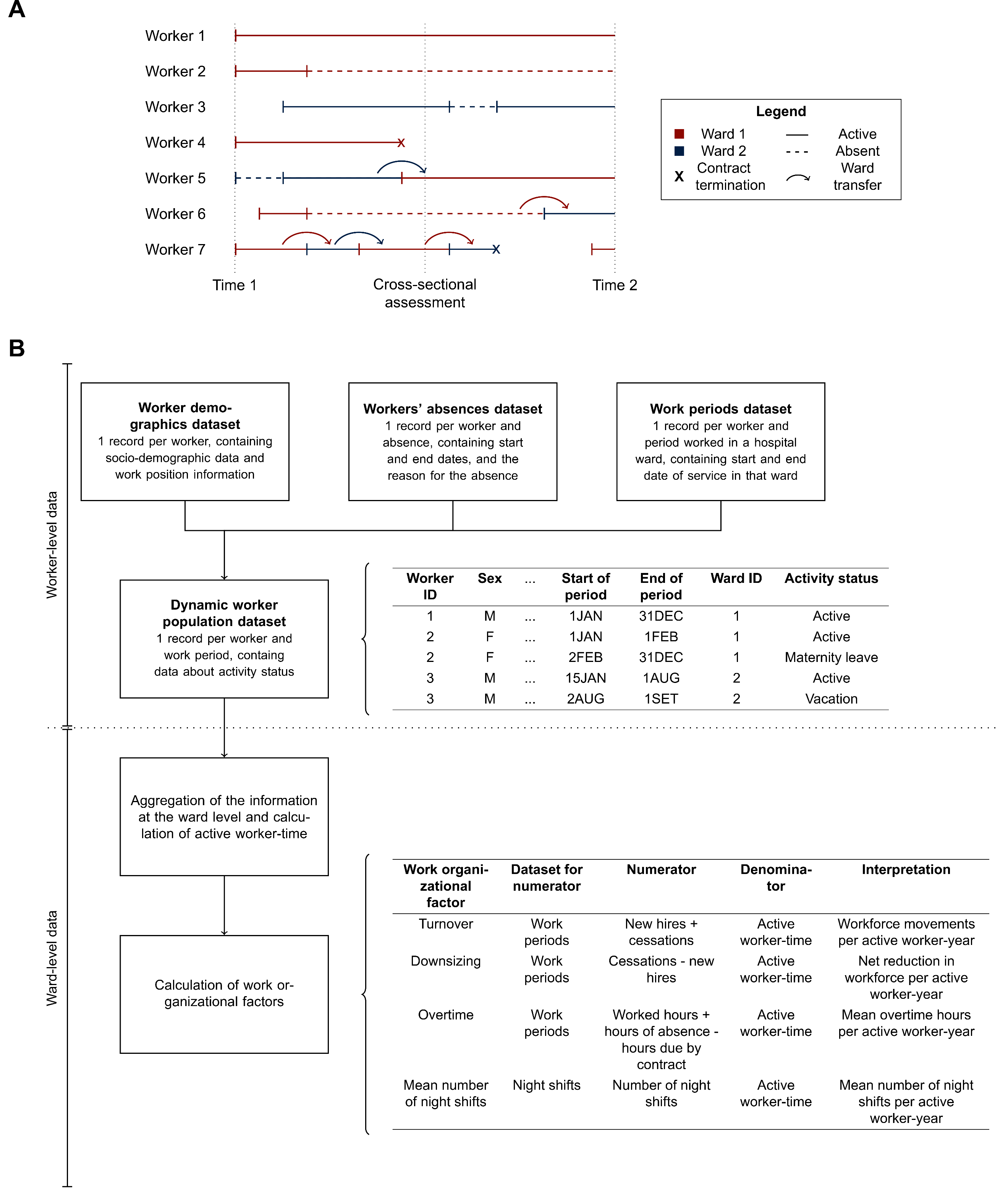
**Figure S1.** Representation of the dynamic nature of the workforce population over time (Panel A) and workflow of the methodology employed to estimate work organizational indicators per ward and year relative to the active workers (Panel B)

**Figure S2.** Distribution of the work organizational factors by number of workplace violence episodes, in wards with night shift scheduling


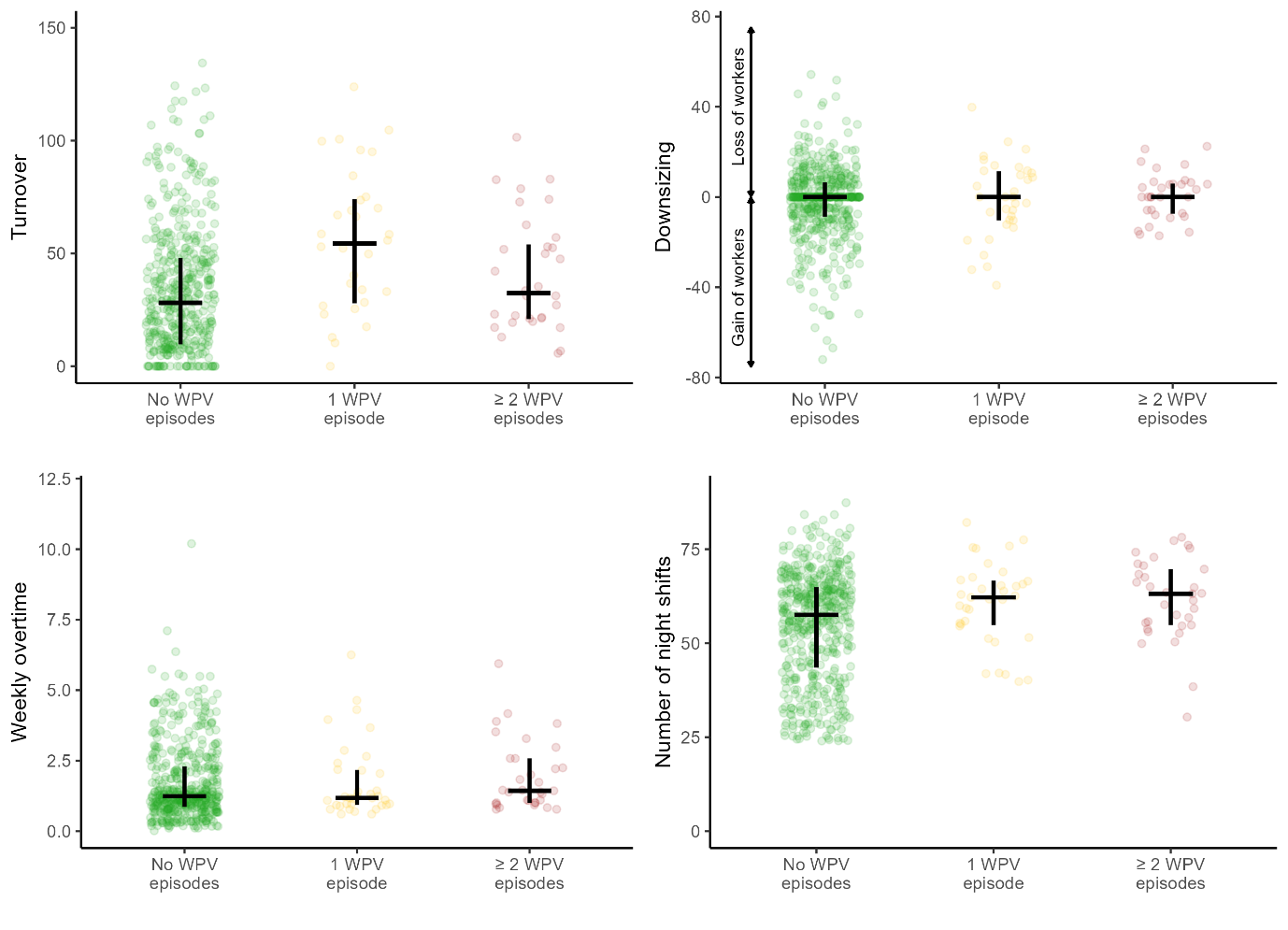


| **Table S1.** Number of registered workers and active worker-time per year | | | | | |
| --- | --- | --- | --- | --- | --- |
| Year | Registered workers | | | Active worker-time^a^ | Ratio of active worker-time to registered at December 31^st^  (%) |
|  | At January 1^st^ | At July 1^st^ | At December 31^st^ |  |  |
| 2016 | 3842 | 3872 | 3904 | 3092.2 | 79.2 |
| 2017 | 3901 | 3975 | 4062 | 3207.6 | 79.0 |
| 2018 | 4067 | 4001 | 4009 | 3243.0 | 80.9 |
| 2019 | 3995 | 4009 | 4081 | 3227.4 | 79.1 |
| 2020 | 4090 | 4098 | 4095 | 3271.9 | 79.9 |
| 2022 | 4107 | 4109 | 4004 | 3249.4 | 81.2 |
| Note. ^a^ The active worker-time can be interpreted as the daily average of active workers over the year | | | | | |

| **Table S2.** Number of active workers, percentage of women, median age and hospital seniority, and work organizational factors, in the wards with regular night shift scheduling during the study years. | | | | | | |
| --- | --- | --- | --- | --- | --- | --- |
|  | **Year (number of wards)** | | | | | |
|  | **2016 (n=101)** | **2017 (n=102)** | **2018 (n=101)** | **2019 (n=90)** | **2020 (n=95)** | **2022 (n=86)** |
| Average number of active workers | 17.0 [10.6, 24.1] | 17.5 [11.6, 25.0] | 17.9 [11.5, 26.1] | 17.3 [11.0, 26.6] | 16.7 [10.0, 27.7] | 17.9 [10.3, 29.2] |
| Percentage of women | 80.4 [66.7, 91.3] | 81.6 [63.9, 89.1] | 80 [65.0, 89.7] | 78.4 [64.6, 91.7] | 78.1 [62.2, 88.6] | 76.6 [63.9, 83.8] |
| Median age | 47.4 [43.2, 49.6] | 47.2 [43.0, 50.5] | 47.8 [42.9, 50.1] | 47.7 [42.8, 50.7] | 45.8 [40.1, 50.0] | 44.9 [38.5, 49.9] |
| Median hospital seniority | 15.1 [ 9.2, 19.1] | 13.2 [ 9.9, 19.3] | 13.2 [ 9.8, 18.6] | 12.3 [ 8.8, 19.5] | 11.6 [ 4.7, 15.6] | 9.5 [ 4.6, 15.1] |
| Turnover, per 100 active workers | 10.7 [ 0.0, 22.9] | 42.2 [21.2, 66.6] | 38.7 [22.2, 57.6] | 21.9 [ 6.8, 37.2] | 25 [11.1, 48.2] | 58.2 [33.7, 99.1] |
| Downsizing, per 100 active workers | 0.0 [-4.9, 5.0] | 0.0 [-10.9, 3.2] | 0.0 [-5.6, 9.5] | 0.0 [-12.8, 5.1] | -2.9 [-15.2, 1.7] | 5.6 [-1.8, 15.3] |
| Weekly overtime, per active worker | 1.0 [0.7, 1.3] | 1.0 [0.7, 1.3] | 1.0 [0.8, 1.4] | 1.0 [0.8, 1.4] | 3.8 [2.6, 4.3] | 2.1 [1.5, 2.9] |
| Mean number of night shifts^a^ | 58.5 [46.6, 65.2] | 57.2 [48.0, 63.8] | 58.4 [47.5, 64.2] | 56.9 [42.3, 67.0] | 59.2 [40.6, 67.4] | 61.8 [50.4, 66.4] |
| Note. Medians [interquartile ranges] are reported, unless otherwise specified  ^a^ per active worker engaged in night shifts | | | | | | |

| **Table S3.** Comparison of work organizational factors by number of workplace violence episodes across ward-year instances | | | |
| --- | --- | --- | --- |
| **Variable** | **No WPV episodes (n=1281)** | **One WPV episode**  **(n=50)** | **Two or more WPV episodes (n=50)** |
| Turnover | 21.0 [ 5.2, 42.5] | 49.8 [25.8, 69.8] | 32.6 [17.7, 56.0] |
| Downsizing | 0.0 [-7.5, 7.5] | 0.0 [-10.3, 9.6] | 0.0 [-5.7, 9.5] |
| Weekly overtime | 1.1 [0.5, 2.2] | 1.2 [0.9, 2.2] | 1.1 [0.8, 2.2] |
| Night shift scheduling^a^, n (%) | 507 (39.6) | 35 (70.0) | 33 (66.0) |
| Note. Medians [interquartile ranges] are reported, unless otherwise specified  Abbreviations: WPV = Workplace violence  ^a^ Average of at least two night shifts per month per active worker engaged in night shifts | | | |
